# Dual roles of endogenous and exogenous galectin-1 in the control of testicular immunopathology

**DOI:** 10.1038/srep12259

**Published:** 2015-07-30

**Authors:** Cecilia V. Pérez, Leticia G. Gómez, Gisela S. Gualdoni, Livia Lustig, Gabriel A. Rabinovich, Vanesa A. Guazzone

**Affiliations:** 1 Instituto de Investigaciones Biomédicas (INBIOMED), Universidad de Buenos Aires (UBA) – Consejo Nacional de Investigaciones Científicas y Técnicas (CONICET), Facultad de Medicina, Ciudad Autónoma de Buenos Aires, Argentina; 2 Laboratorio de Inmunopatología, Instituto de Biología y Medicina Experimental (IBYME), CONICET, Ciudad Autónoma de Buenos Aires, Argentina; 3 Facultad de Ciencias Exactas y Naturales, UBA, Ciudad Autónoma de Buenos Aires, Argentina

## Abstract

Galectin-1 (Gal-1), a proto-type member of galectin family, is highly expressed in immune privileged sites, including the testis. However, in spite of considerable progress the relevance of endogenous and exogenous Gal-1 in testis pathophysiology have not yet been explored. Here we evaluated the *in vivo* roles of Gal-1 in experimental autoimmune orchitis (EAO), a well-established model of autoimmune testicular inflammation associated with subfertility and infertility. A significant reduction in the incidence and severity of EAO was observed in mice genetically deficient in Gal-1 (*Lgals1*^*−/−*^) versus wild-type (WT) mice. Testicular histopathology revealed the presence of multifocal testicular damage in WT mice characterized by an interstitial mononuclear cell infiltrate and different degrees of germ cell sloughing of seminiferous tubules. TUNEL assay and assessment of active caspase-3 expression, revealed the prevalence of apoptotic spermatocytes mainly localized in the adluminal compartment of seminiferous tubules in EAO mice. A significant increased number of TUNEL-positive germ cells was detected in EAO testis from WT compared with *Lgals1*^*−/−*^ mice. In contrast, exogenous administration of recombinant Gal-1 to WT mice undergoing EAO attenuated the severity of the disease. Our results unveil a dual role of endogenous versus exogenous Gal-1 in the control of autoimmune testis inflammation.

Galectins a family of glycan-binding proteins are mainly defined by a common structural fold and a conserved carbohydrate recognition domain (CRD) of about 130 amino acids that recognizes *N*- and *O*-glycans expressing the disaccharide N-acetyllactosamine [Galβ(1–4)-GlcNAc or LacNAc]^1^. Galectin-1 (Gal-1), a one-CRD member of the galectin family, is secreted to the extracellular milieu through a non-classical endoplasmic reticulum-Golgi-independent pathway^2^. Through its ability to recognize specific glycan structures, Gal-1 influences a variety of physiologic and pathologic processes including pathogen recognition, immune cell signaling, activation and homeostasis, maintenance of placental immune privilege and suppression of autoimmune pathology^3^. Recently, Gal-1 has emerged as a novel hypoxia-regulated pro-angiogenic factor, which controls tumor progression^4^,^5^,^6^ and contributes to the pathogenesis of endometriosis and pre-eclampsia^7^,^8^. Interestingly, Gal-1 expression is regulated throughout the spermatogenic process^9^. This lectin is expressed in Sertoli cells mainly at stages X–II of the spermatogenic cycle and is up-regulated during spermiation (stages VI–VIII) within the luminal pole of seminiferous epithelium, localized on apical stalks of Sertoli cells, on heads of mature spermatids and on bodies of residual cytoplasm. Following spermiation (stage VIII), Gal-1 expression is restored at the basal portion of Sertoli cells and progressively spread out through the whole cells as differentiation of germinal cells proceeded^9^. Moreover, a strong Gal-1 immunoreactivity is also detected in the cytoplasm of Sertoli cells of human testis^10^,^11^. However, in spite of considerable progress, the function of Gal-1 in immune privilege and testis immunopathology has not yet been studied.

Immune privilege implies a special status conferred to some mammalian tissues, where allo- or self-antigens are well-tolerated and inflammation is circumvented^12^. The testis is considered an immune privileged site as it is able to tolerate self-antigens from developing germ cells that appear at puberty long after the establishment of immunocompetence, thus contributing to protect the highly specialized process of spermatogenesis. Multiple mechanisms have been proposed to prevent autoimmune inflammation in the testis including: a) an immunological blood testis barrier (BTB), a structure that reduces the access of germ cell antigens to interstitial immune cells and the passage of antibodies from interstitium to tubular lumen; b) the secretion of numerous immunosuppressive factors mainly by macrophages, Sertoli, peritubular and Leydig cells resulting in a protective local microenvironment against immunologic attack; and c) the presence of regulatory CD4^+^ and CD8^+^ T cells (Tregs) which serve as local suppressors of effector T cell responses^13^. In addition, Sertoli cells can endocytose and degrade apoptotic germ cells and residual bodies, thus preventing potential autoimmune responses against spermatogenic cells^14^,^15^,^16^.

Early experimental data showed that testes can sustain foreign grafts for extended time periods without evidence of rejection^17^. Moreover, allogenic testes ectopically transplanted under the kidney capsule resisted rejection in the absence of generalized immunosuppression^18^,^19^. More recently, co-implantation with Sertoli cells led to prolonged survival of allogeneic pancreatic islets in diabetic mice^20^,^21^. Moreover, Dal Secco *et al*. showed that Sertoli cells can suppress CD8^+^ T cell proliferation through PD-L1-inducing Treg cells^22^. However, in spite of its immune privileged status, the testis can orchestrate both innate and adaptive immunity and promote inflammatory responses to local and systemic infections. The delicate equilibrium between immune privilege and inflammation is modulated by critical cytokines that contribute to ignite immunosuppressive or inflammatory circuits in local or systemic microenvironments. Disruption of these immunoregulatory circuits may lead to the development of autoimmune orchitis and impairment of normal fertility^23^.

Experimental autoimmune orchitis (EAO) is a useful model to study organ-specific autoimmunity and chronic testicular inflammation. Current information on the mechanisms underlying testicular pathology and potential treatments stems from the pioneering work by Tung *et al*.^24^ in mice and the subsequent establishment of the EAO model in rats^13^,^25^. In both models testicular histopathology is characterized by interstitial mononuclear cell infiltrates and damage of seminiferous tubules that exhibit germ cell apoptosis and severe cell sloughing (mainly spermatocytes and spermatids). Progression of EAO is associated with fibrosis, testicular atrophy and infertility^26^.

Here we aimed to investigate the pathophysiologic relevance of endogenous and exogenous Gal-1 in testicular immune privilege and pathology using the EAO model.

## Materials and Methods

### Animals

Six- to eight-week-old male C57BL/6J (wild-type, WT) mice were purchased from the animal facilities at the Facultad de Ciencias Veterinarias (Universidad Nacional de La Plata, Argentina). Mice with equivalent genetic background and mutation in the Gal-1-encoding gene (*Lgals1*^*−/−*^) were originally supplied by F. Poirier (Jacques Monod Institut, Paris). Animals were kept at 22 °C with a 14 h light-10 h dark schedule and fed standard food pellets and water *ad libitum*. All experimental protocols were performed in accordance with approved guidelines from the Universidad de Buenos Aires (UBA) and the Instituto de Biología y Medicina Experimental (IBYME, CONICET).

### Induction of EAO

*Lgals1*^*−/−*^ and WT mice were actively immunized with mouse testicular homogenate (TH) (experimental group; E) as previously described^27^. Briefly, 300 mg of dry weight TH in 1.5 ml of distilled water was emulsified with an equal volume of Freund’s adjuvant prepared by adding 100 mg of *Mycobacterium tuberculosis* (H37Ra, Difco Laboratories, Detroit, Michigan, USA) to incomplete Freund’s adjuvant (Sigma-Aldrich, St. Louis, MO, USA). Each animal received 0.1 ml of the emulsion distributed in one hind footpad, base of tail and hind flank by s.c. injection. In addition, each animal received 0.4 μg of Pertussis toxin (Ptx; Sigma-Aldrich) dissolved in 0.1 ml of saline solution following by an additional dose 24 h later by i.p. injection. Control (C) mice received adjuvants and Ptx, but no TH following the same scheme. Normal (N) untreated mice were also studied. All animals were killed 30 days after immunization. Testes were removed and processed for different experimental settings as detailed below.

### Histopathology

Histopathology of the testis and epididymis was evaluated in paraffin-embedded Bouin’s-fixed sections obtained from three different levels and stained with hematoxylin-eosin or PAS-hematoxylin. To quantify EAO incidence and severity, we used an established score^28^. Briefly, EAO score was graded by evaluation of seminiferous tubules, straight tubules, epididymis and vas deferens inflammation, seminiferous tubules germ cell loss, and epididymis and vas deferens sperm depletion. Moreover, testis and epididymal inflammation scores were determined as follows: 1, focal; 5, diffuse inflammation with necrosis; and 2–4, range of incremental inflammation. Aspermatogenesis: 1–10 represent the percentage of seminiferous tubules with reduction or absence of germ cells. Maximum EAO score is 31. Animals with a score under or equal to 5 were considered free of orchitis. Quantification of blood vessels was performed by light microscopy with a 10x ocular and a 40x objective. Data were obtained from 6 mice/group. Two sections/mouse were counted and results were expressed as the number of vessels per 100 seminiferous tubules cross-sections. No distinction was drawn between arterioles, venules and capillaries.

### Immunohistochemistry

Immunoperoxidase staining was performed using the avidin-biotin system (ABC Vectastain Kit, Vector Lab., Burlingame, CA, USA) to detect Gal-1 or active caspase-3. Deparaffinized and hydrated sections were subjected to antigen retrieval by microwaving (370 W) in 0.01 M citrate buffer, pH 5.5 for 15 min. Endogenous peroxidase activity was blocked by treatment with 0.3% H_2_O_2_ in methanol for 30 min. Sections were washed in phosphate buffered saline (PBS) and incubated with 5% skim milk, 0.01% Triton X-100 to detect Gal-1 or with 0.1% saponin in PBS 0.01% Triton X-100 to detect active caspase 3. Sections were then incubated with avidin/biotin blocking solution (Vector Lab.). After incubation with a rabbit anti-Gal-1 polyclonal antibody (1:50) as described [4] or anti-human/mouse active caspase-3 antibody (10 μg/mL) (R&D Systems, Minneapolis, MN, USA) for 48 h at 4 °C in a humidified chamber, sections were incubated with biotinylated anti-rabbit IgG (1:375, Vector Lab.). Then the reaction was amplified using ABC Vectastain kit and the 3-3’diaminobenzidine-H_2_O_2_ (DAB Substrate Kit, Vector Lab.) was used as peroxidase substrate. Sections were counterstained with hematoxylin. Negative controls were obtained by incubating sections with the corresponding IgG isotypes instead of primary antibodies.

### Western blot

Expression of Gal-1 protein was evaluated in the seminiferous tubule and interstitial cell (IC) fractions. Briefly, testes were decapsulated and placed on a Petri dish containing PBS. Seminiferous tubules (ST) were mechanically separated from IC with needles and filtered through a fine stainless steel screen. The cell suspension, containing IC, was centrifuged at 300 × g for 10 min. Both fractions were homogenized in ice-cold lysis buffer [50 mM Tris-HCl (pH 7.4), 150 mM NaCl, 2 mM EDTA, 0.1% SDS, 0.5% sodium desoxycholate, 1% NP-40] with protease inhibitors (2 mM phenylmethylsulphonyl fluoride, 10 μg⁄ml leupeptin, 10 μg⁄ml pepstatin A and 10 μg⁄ml aprotinin; Sigma-Aldrich). Homogenates were centrifuged at 13,500 g for 30 min at 4 °C. Proteins were measured in supernatants using the Bio-Rad DC Protein Assay (Bio-Rad Laboratories, Hercules, CA, USA). Equal amounts of protein (25 μg from the ST fraction or 100 μg from the IC fraction) were resolved in a 15% SDS-polyacrylamide gel electrophoresis. Proteins were electroblotted at 150 V for 60 min to PVDF membranes (Bio-Rad Laboratories). Transfer was monitored by Ponceau S staining. Membranes were blocked with 5% non-fat dry milk in TBS containing 0.1% Tween 20 for 1 h. Blots were probed overnight with a rabbit anti-Gal-1 polyclonal antibody (1:5000) generated and used as described [4] followed by anti-actin (1:3000; Sigma-Aldrich) polyclonal antibody. Blots were washed and incubated with a biotinylated goat anti-rabbit IgG (1:6000; Vector Lab.) followed by streptavidin-horseradish peroxidase conjugates (Chemicon International Inc, Millipore Co., Billerica, MA, USA). Proteins were visualized by enhanced chemiluminescence. Images were captured using the GeneSnap software (7.08.01 version) and were analyzed with Gene Tools software (4.01.02 version) from SynGene (Synoptics Ltd, Frederick, MD, USA).

### TUNEL assay

Deparaffinized and hydrated sections from testes were irradiated in a microwave oven (370 W for 5 min) in 10 mM sodium citrate buffer, pH 6 and permeabilized with 0.1% Triton X-100 in 0.1% sodium citrate for 5 min at 4 °C. Non-specific labeling was prevented by incubating the preparations with blocking solution (5% blocking reagent; Roche Molecular Biochemicals GmbH, Mannheim, Germany, in 150 mM NaCl and 100 mM maleic acid, pH 7.5) for 30 min at room temperature. The apoptotic DNA was 3´-end labeled with digoxigenin-11-dideoxyuridine triphosphate (Dig-11-ddUTP; Roche) by the TdT reaction (0.17 U/ml TdT; Roche) in TdT buffer for 1 h at 37 °C. In negative controls, TdT enzyme was replaced with the same volume of distilled water. Preparations were then incubated with blocking solution (2% blocking reagent in 150 mM NaCl and 100 mM maleic acid, pH 7.5) for 30 min at room temperature, followed by the detection of the Dig-11-ddUTP with alkaline phosphatase-conjugated anti-digoxigenin antibody (1:2000; Roche) for 2 h at room temperature. Sections were rinsed and equilibrated in alkaline phosphatase buffer (100 mM Tris–HCl, 100 mM NaCl, 50 mM MgSO_4_, pH 9.5) containing 1 mM levamisole (Sigma-Aldrich). Then, alkaline phosphatase substrates, nitroblue tetrazolium and 5-bromo-4-chloro-3-indolyl-phosphate (NBT/BCIP; Roche) were added for 60 min. The reaction was stopped by washing preparations with TE buffer (10 m MTris–HCl, 1 mM EDTA, pH 8.0). Sections were counterstained with eosin, dehydrated and mounted. Finally, TUNEL-positive cells were quantified in 100 tubules per testis.

### Experimental design: administration of rGal-1

Production and purification of recombinant Gal-1 (rGal-1) was performed as outlined previously^29^. Mice (WT) from E group were injected i.p. with rGal-1 (2.5 mg/kg) or saline solution (vehicle). Treatment started 21 days after immunization and continued throughout the experiment with intervals of 2 days. Mice were killed at 30 days after immunization. Testes were removed and processed for histopathology.

### Statistical analysis

Comparisons between groups were assessed by the non-parametric Mann-Withney *U* test or Kruskal–Wallis One-Way ANOVA. *P* values less than 0.05 were considered significant.

## Results

### Histopathologic features of the EAO mouse model

**Figure 1:**
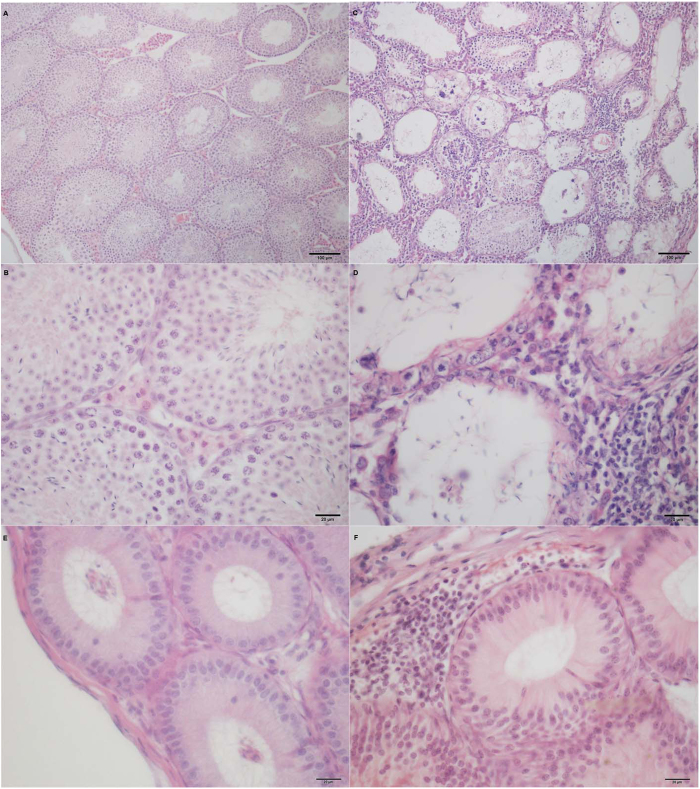
Testicular and epididymal histopathology. EAO was induced in WT (C57BL/6J) mice by immunization with testicular antigens and adjuvants. Testis (**C** and **D**) and epididymis (**F**) sections from the experimental group sacrificed 30 days after immunization exhibited an interstitial inflammatory cell infiltrate. Severe testicular damage was characterized by germ cell sloughing of the seminiferous tubules. Note the severe tubular atrophy showing decreased diameter of seminiferous tubules in experimental group. Testis (**A** and **B**) and epididymis (**E**) section from control group show normal histological features. **H**&**E**.

To understand the relevance of endogenous Gal-1 in testicular immunopathology, we first sought to establish an EAO model in C57BL/6J mice. Notably, 95.5% of WT mice from experimental group sacrificed 30 days after the immunization developed autoimmune orchitis with different degrees of severity. Testicular pathology was characterized by an interstitial inflammatory cell infiltrate and damage of the germinal epithelium (Fig. 1C,D). Immune cell infiltrate was mostly confined to the subalbuginea interstitium and extended through the whole organ in severe EAO. Seminiferous tubules showed the presence of degenerating spermatocytes, multinucleated germ cells and different degrees of germ cell sloughing in the lumen. In severe EAO only spermatogonia, few spermatocytes and Sertoli cells remained attached to the tubular wall. Vacuolization of Sertoli cell cytoplasm was frequently observed. In the epididymis interstitial immune cell infiltrates (F) and the presence of immature germ cells in tubular lumen were frequently detected in EAO mice. Occasionally, immune cell infiltrates were observed within damaged seminiferous tubules. No pathological alterations were found in the testis and epididymis of normal and control groups (Fig. 1A,B,E).

Assessment of the number of testicular vessels revealed a significant increase of these structures in the testicular interstitium of mice with orchitis. Interestingly, the extent of vascularization was associated with the degree of histopathologic damage (Normal (N) score: 129.90 ± 9.90, Control (C) score: 113.00 ± 5.35, Experimental (E) with mild EAO score: 164.50 ± 8.86^a,b^, E with severe EAO score: 196.30 ± 6.31^c,d^. ^a,b^p < 0.05 vs N and C, ^c,d^p < 0.001 vs N and C).

### Regulated expression of Gal-1 in inflamed testis

**Figure 2:**
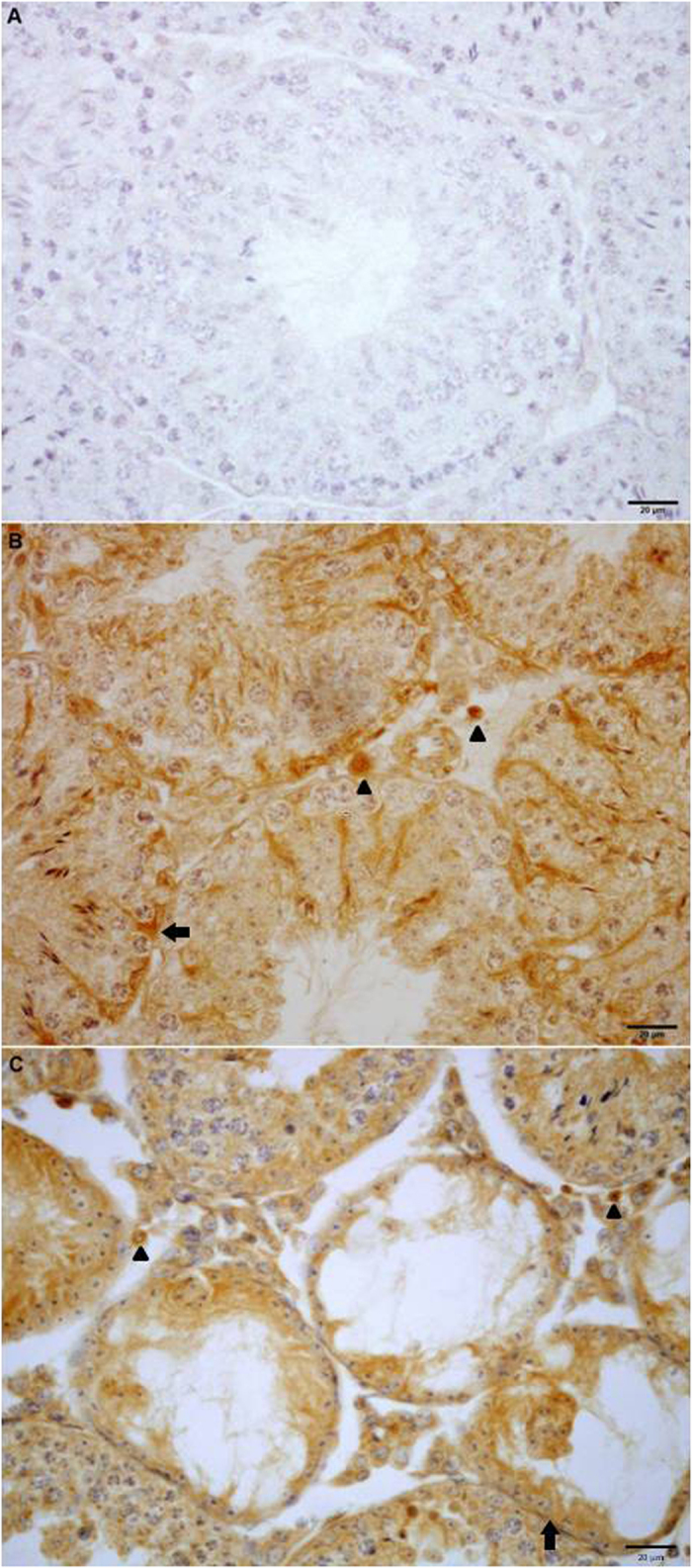
Immunohistochemistry of Gal-1 expression in mouse testis sections. Photomicrographs of paraffin-embedded sections of normal (**B**) and experimental (**C**) adult testes immunostained with Gal-1 antibody. Strong Gal-1 immunoreactivity can be observed within the seminiferous tubules associated with both Sertoli cells (arrow) and differentiating germ cells and also in Leydig cells (arrowhead) located in the interstitial spaces. Omission of primary anti-Gal-1 antibody was used as a negative control (**A**).

**Figure 3:**
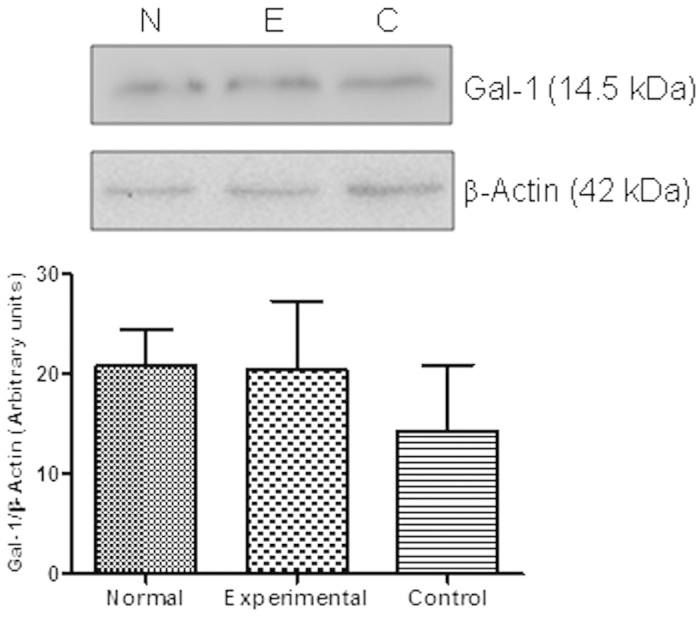
Expression of Gal-1 by interstitial cells of normal (N), control (C) and experimental (E) mice was analyzed by Western blot. The blots were cropped, and the full-length blots are presented in the supplementary information (Fig. S1 online). No changes in Gal-1 expression were observed between different groups. Each bar represents the mean ± SEM of 6 animals.

**Figure 4:**
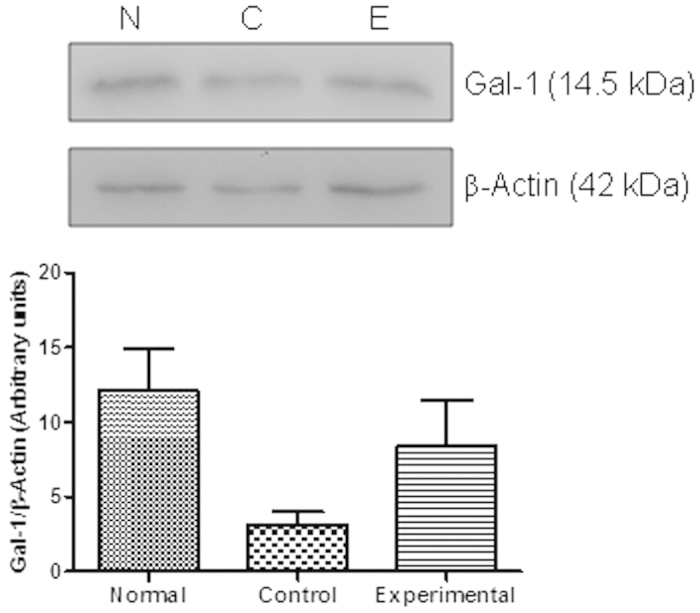
Expression of Gal-1 by seminiferous tubules of normal (N), control (C) and experimental (E) mice was analyzed by Western blot. The blots were cropped, and the full-length blots are presented in the Supplementary Information (Fig. S2 online). No changes in Gal-1 expression were observed among different groups. Each bar represents the mean ± SEM of 6 animals.

To analyze the regulated expression of Gal-1 in inflamed testis, sections from normal and EAO mice were processed by immunohistochemistry. In testicular interstitium from normal mice, Gal-1 was detected in Leydig cells, as well as in the seminiferous tubules, in Sertoli cell cytoplasm and germ cells. A high Gal-1 expression was detected in the entire cytoplasm of Sertoli cells from the basal to the apical area. A milder staining was observed in differentiating germ cells and spermatozoa. A similar localization and intensity of Gal-1 expression was detected in normal and EAO mice. No staining was observed in sections incubated with IgG isotype control antibody instead of primary antibody (Fig. 2). No changes in Gal-1 expression were detected by Western blot in interstitial cells (Fig. 3) and in seminiferous tubule (Fig. 4) fractions from normal, control and EAO group.

### Lack of Gal-1 reduces the incidence and severity of EAO

**Figure 5:**
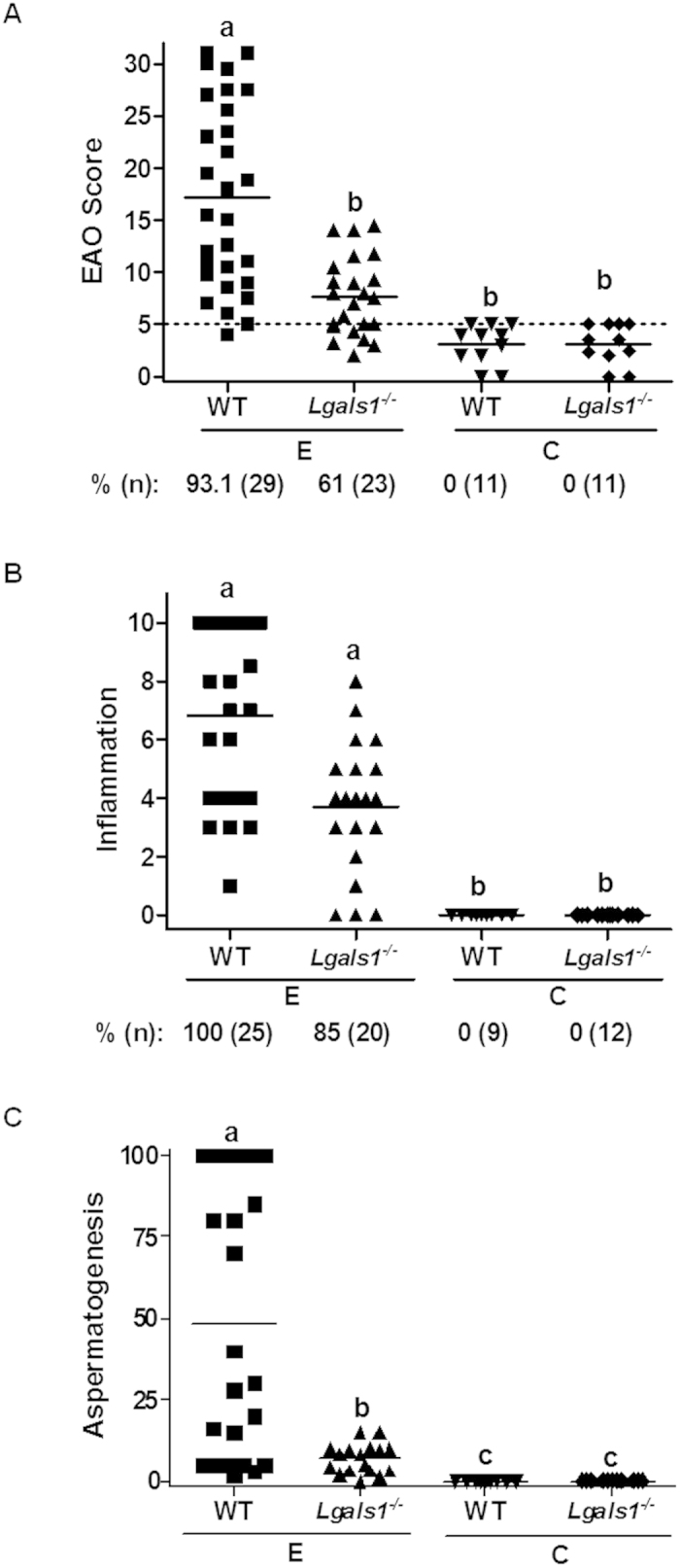
Impact of endogenous Gal-1 in EAO incidence and severity. **A-** A significant decrease in EAO incidence (%) and reduced severity of the disease was observed in *Lgals1*^*−/−*^ versus WT mice. EAO score from WT, *Lgals1*^*−/−*^ experimental (**E**) group and control (**C**) group mice assessed by evaluation of seminiferous tubules, straight tubules, epididymis and vas deferens, germ cell loss, inflammation and sperm depletion. Animals with a score below or equal to 5 (…) were considered free of orchitis. **B-** Inflammation represents the degree of cellular infiltrate in the testicular interstitium and surrounding straight tubules. %: percentage of inflammation. Animals with an inflammation value equal to 0 were considered without inflammation. **C-** Aspermatogenesis represents the percentage of seminiferous tubules with reduction or absence of germ cells. Horizontal lines represent the mean value. Each symbol represents a single mouse. Values with different letter superscripts differ significantly (p < 0.05).

The role of endogenous Gal-1 was studied *in vivo* in the EAO model. EAO was induced in male WT and *Lgals1*^*−/−*^ mice as described in *Materials and Methods*. Whereas 93.1% of WT mice developed orchitis, a significant decrease in EAO incidence (61%) was observed in mice devoid of Gal-1. Interestingly, *Lgals1*^*−/−*^ mice exhibited a significant reduction of EAO severity compared to their WT counterpart (Fig. 5A), showing focal testicular damage with mild cell infiltrate. Moreover, a significant lower percentage of seminiferous tubules with germ cell sloughing was also observed in *Lgals1*^*−/−*^ mice (Fig. 5C). Interestingly, a trend toward a reduction of testis inflammation was also observed in *Lgals1*^*−/−*^ mice, although these data were not found to reach statistical significance (Fig. 5B). Of note, WT and *Lgals1*^*−/−*^ mice from control group did not develop orchitis (Fig. 5A). Thus, in contrast to the higher pathology observed in the context of Gal-1 deficiency in other experimental autoimmune models including experimental autoimmune encephalomyelitis (EAE)^30^ and collagen-induced arthritis (CIA)^31^, EAO pathology was found to be less severe in *Lgals1*^*−/−*^ compared to WT mice, suggesting context-dependent Gal-1-mediated regulation of autoimmune pathology.

### Impact of endogenous Gal-1 on germ cell apoptosis

**Figure 6:**
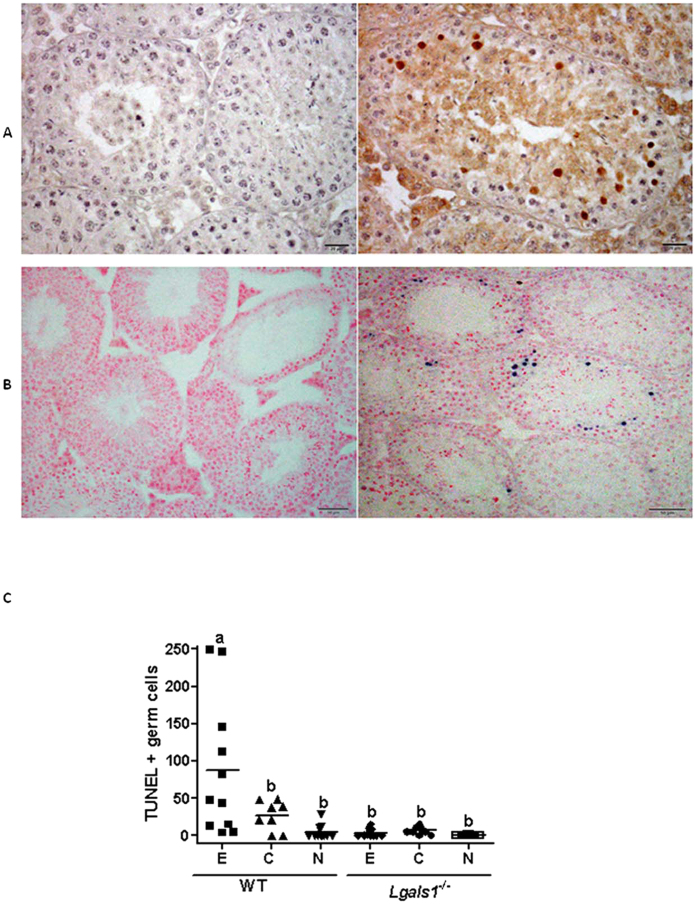
Impact of endogenous Gal-1 on germ cell apoptosis during the course of EAO. Testes from experimental (**E**), control (**C**) or normal (N) WT and *Lgals1*^*−/−*^ group mice were removed at 30 days after immunization, paraffin-embedded, and processed for caspase-3 expression and *in situ* TUNEL assay. **A-** Representative microphotograph (right picture) shows expression of active caspase-3 protein by immunoperoxidase staining in testis section from EAO WT mice group. Spermatocytes are immunoreactive for caspase-3. Omission of primary antibody shows negative staining in left picture. **B-** Representative microphotographs of TUNEL assay performed in EAO testis sections from WT (right picture) and *Lgals1*^*−/−*^ (left picture) mice stained with eosin. TUNEL-positive cells are stained in blue. **C-** Quantitative assessment of TUNEL-positive cell number per 100 tubules per testis evaluated in a blinded fashion in each group. Horizontal lines represent the mean value and each symbol represents a single mouse. Values with different letter superscript differ significantly (p *< *0.05).

To examine the mechanisms underlying Gal-1 control of EAO severity, we next evaluated the impact of endogenous Gal-1 in germ cell apoptosis. For this, we studied the expression of the active form of caspase-3 and quantified the number of apoptotic germ cells using an *in situ* TUNEL assay. We found that, spermatocytes from testis of mice with EAO were immunoreactive for caspase-3 (Fig. 6A) and positive for TUNEL assay (Fig. 6B). However, the number of apoptotic germ cells per seminiferous tubule in EAO WT mice was significantly higher than control and normal WT as well as experimental, control and normal *Lgals1*^*−/−*^ groups (Fig. 6C). Of note, data from *Lgals1*^*−/−*^ mice were comparable among experimental, control and normal groups (Fig. 6C).

### Administration of exogenous rGal-1 reduces the severity of EAO

**Figure 7:**
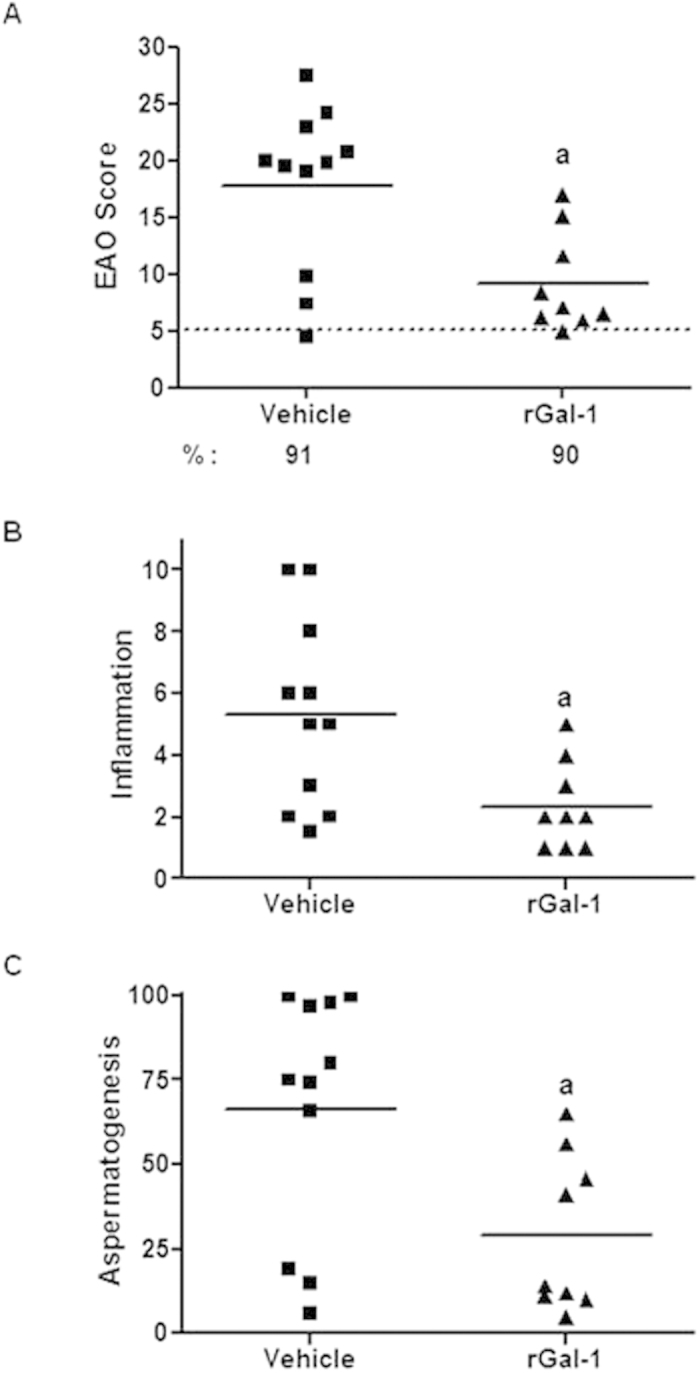
Impact of exogenous Gal-1 in testicular immunopathology. **A-** Administration of rGal-1 reduces the severity of EAO. EAO score of mice immunized with TH and adjuvants and injected with saline solution (vehicle) or rGal-1. Score evaluated seminiferous tubules, straight tubules, epididymis and vas deferens and includes germ cell loss, inflammation and sperm depletion. Animals with a score below or equal to 5 (…) were considered free of orchitis. **B-** Inflammation represents the degree of cellular infiltrates in the testicular interstitium and surrounding straight tubules. %: percentage of inflammation. Animals with an inflammation value equal to 0 were considered to be devoid of inflammation. **C-** Aspermatogenesis represents the percentage of seminiferous tubules with reduction or absence of germ cells. Horizontal lines represent media value. Each symbol represents a single mouse. (^a^p < 0.05 vs vehicle).

**Figure 8:**
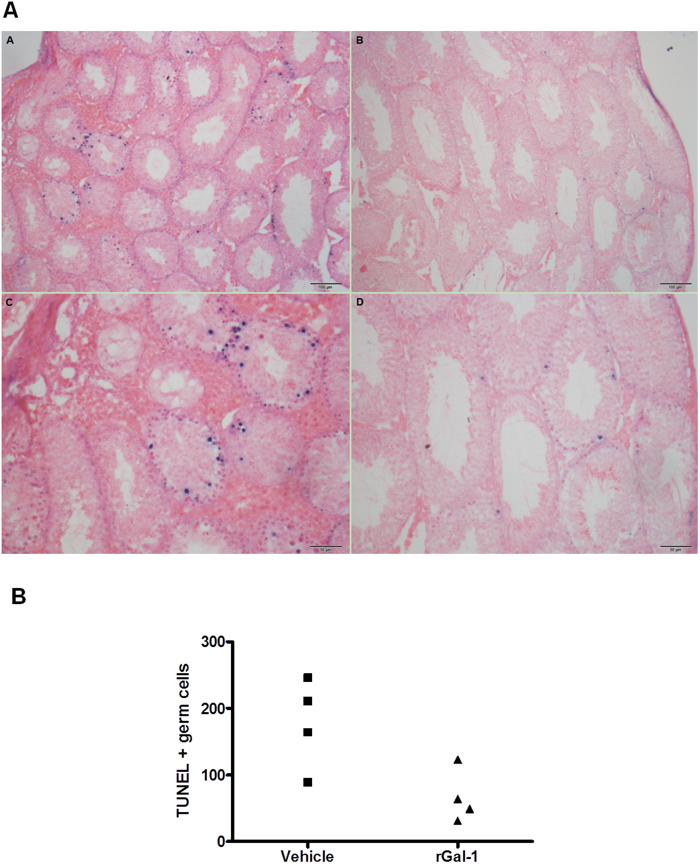
Impact of exogenous Gal-1 on germ cell apoptosis. Testes from mice immunized with TH and adjuvant and injected with saline solution (vehicle) or recombinant Gal-1 (rGal-1) were removed, paraffin-embedded, and processed by *in situ* TUNEL assay. **A-** Representative microphotographs of TUNEL assay performed in EAO testis sections from mice treated with vehicle (**A** and **C**) or rGal-1 (**B** y **D**) and counterstained with eosin. TUNEL-positive cells are stained in blue. **B-** Quantitative assessment of TUNEL-positive cell number in 100 tubules per testis evaluated in a blinded fashion in each group. Each symbol represents a single mouse. (p = 0.05).

Based on the immune-inhibitory activity of exogenous rGal-1 in different models of autoimmune pathology^30^,^32^,^33^,^34^,^35^, we evaluated the effects of rGal-1 administration in WT mice during the course of EAO. Notably, injection of rGal-1 starting 21 days after mice immunization significantly reduced severity of EAO. Interestingly, testis inflammation and seminiferous tubule germ cell loss were both reduced in mice treated with rGal-1 (Fig. 7). TUNEL assay revealed that only germ cells underwent apoptosis (Fig. 8A). Quantitative assessment of TUNEL-positive cells revealed that administration of rGal-1 in WT mice undergoing orchitis reduced the number of apoptotic cells compared with vehicle-treated mice. (Figure 8B) Although we could not observe TUNEL-positive infiltrating immune cells, we cannot rule out an immune inhibitory mechanism mediated by T cell apoptosis in associated secondary lymphoid organs as demonstrated in other experimental models^30^,^32^,^33^,^34^.

Thus, exogenous Gal-1 suppresses disease severity and inflammation and protected germ cells from inflammation-induced apoptosis. Collectively, these results highlight the dual and paradoxical roles of endogenous versus exogenous Gal-1 in the control of testicular immunopathology.

## Discussion

Compelling evidence on the immunoregulatory activity of Gal-1 and its abundant expression in immune privileged tissues including placenta, ovary and testis^3^, suggested that this endogenous lectin could play a critical role in the homeostatic control of immune privilege and inflammation in these tissues. We undertook this study to examine the regulated expression of Gal-1 in normal and inflamed testis and its ability to control testicular pathology.

Previous work documented the regulated expression of Gal-1 in rat testicular somatic and germ cells under physiologic conditions^9^. These early studies hypothesized that Gal-1 might confer immune privilege to the testis by providing Sertoli cells with a mechanism to selectively eliminate infiltrating T lymphocytes. Seeking to understand the role of endogenous Gal-1 in testis homeostasis and inflammation, we assessed the expression and localization of Gal-1 in normal versus inflamed testis and evaluated the ability of WT and *Lgals1*^*−/−*^ mice to develop autoimmune orchitis after active immunization with sperm antigens and adjuvants.

First, we confirmed that Gal-1 was expressed by Sertoli, Leydig and germ cells of adult mice as it has been previously demonstrated in rat, human and mice testis^9^,^11^,^36^. In Sertoli cells, Gal-1 mRNA and protein expression was found to be differentially regulated during spermatogenesis, suggesting that the Gal-1 function in the adult seminiferous epithelium could depend on its cellular and subcellular distribution at different stages of the spermatogenic cycle^36^. Interestingly, we found comparable expression levels of Gal-1 in normal and EAO mice suggesting that testis inflammation was not associated with an upregulation of Gal-1 expression. This result also differs from data reported in other autoimmune models of inflammation including CIA^31^ and EAE^29^,^37^ where substantial upregulation of Gal-1 was observed during the peak of resolution of autoimmune pathology.

Through binding and cross-linking specific cell surface glycans, Gal-1 functions as a homeostatic immune inhibitory signal that blunts pathogenic Th1 and Th17 responses^30^, favors the differentiation of FoxP3^+^ Treg cells^38^,^39^ and instructs dendritic cells to become tolerogenic^37^. These glycosylation-dependent functions account for the capacity of this lectin to dampen inflammation in autoimmune and chronic inflammatory disorders and to favor immune escape in different types of tumors^3^.

Based on the known immunosuppressive activities of Gal-1, we hypothesized that mice deficient in Gal-1 would develop more severe autoimmune orchitis compared to WT mice. Surprisingly, the incidence and severity of EAO were lower in *Lgals1*^*−/−*^ versus WT mice. This effect contrasted with the well established role of endogenous Gal-1 in restraining chronic inflammation in other models including EAE^30^ and CIA^31^, suggesting that the impact of endogenous Gal-1 in autoimmune inflammation may be context-dependent and/or that Gal-1 regulation of germ cell fate may prevail over the effects on T-cell dependent inflammation. Thus, endogenous Gal-1 may act either by increasing the inflammatory response or by inducing germ cell apoptosis.

Similar to our findings, Bischoff *et al*. observed that mice lacking Gal-1 developed a reduced inflammatory response mediated by glial cells in an experimental model of epileptic seizures^40^. Furthermore, Iqbal *et al*. showed different effects of this lectin in a mouse carrageenan paw edema model in which the inflammatory response presents an acute and a delayed phase^41^. In *Lgals1*^*−/−*^ mice, the acute phase followed a similar inflammatory profile compared to WT mice; however, a blunted response was observed for the second phase with significantly lower edema values and decreased immune cell number. Interestingly, the same authors^31^ reported a critical inhibitory role for endogenous Gal-1 during the development of chronic inflammation in the CIA model, suggesting that Gal-1 could play different roles whether it acts early or late during the course of acute or chronic inflammation. Moreover, these responses could be influenced by cellular and molecular networks operating in inflammatory and tissue microenvironments, including other immune-inhibitory pathways (e.g. PD-1/PD-L1 axis, Fas/Fas ligand pathway or secretion of immunosuppressive TGF-β or IL-10) which may act in concert to regulate tissue homeostasis. Of note, we could find no significant differences in the frequency of CD8^+^ T cells, CD11b^+^ macrophages and FoxP3^+^ regulatory T cells among different experimental groups (data not shown) suggesting that, in this particular experimental model, a direct pro-apoptotic effect on germ cells prevails over other regulatory or tolerogenic circuits. Although this is the first study demonstrating the effects of endogenous versus exogenous Gal-1 in regulating testicular immunopathology, further work is warranted at elucidating in depth the molecular mechanisms underlying these biological effects.

An extensively documented function of galectins is the control of immune and epithelial cell survival^42^,^43^. Whereas some galectins display pro-apoptotic activity, others function by protecting cells from common pro-apoptotic stimuli. Interestingly, exogenous galectins can trigger pro-apoptotic or anti-apoptotic events through binding to cell surface glycoproteins, whereas intracellular galectins control cell fate through interaction with endogenous signaling pathways^44^. Sensitivity of immune cells to Gal-1-induced cell death has been shown to be regulated by the glycosylation state of the cells and involves Fas-dependent or -independent pathways^30^,^43^,^45^,^46^.

Regarding the impact of Gal-1 on non-immune cells, Bischoff *et al*. reported a selective pro-apoptotic role of Gal-1 toward a subpopulation of forebrain neurons in an experimental model of epileptic seizures induced by pilocarpine injection in adult mice^40^. Moreover, Muglia and colleagues showed that endogenous Gal-1 regulates small intestine villi length through mechanisms involving modulation of epithelial cell death^43^. Furthermore, *in vitro* studies showed that Gal-1 induces apoptosis of rat testis Leydig cells through interactions with extracellular matrix proteins^47^. Our results demonstrate that Gal-1 acts by inducing apoptosis of germ cells. A significant increase of TUNEL-positive germ cells was detectable in the testis of WT compared to *Lgals1*^*−/−*^ mice or to normal untreated mice. Thus, Gal-1-induced germ cell apoptosis may account for the augmented susceptibility and severity of orchitis in WT versus *Lgals1*^*−/−*^mice. However, we cannot rule out the possibility that other Gal-1-mediated functions may contribute to this effect. Notably Gal-1 also plays a relevant role in angiogenesis by controlling endothelial cell signaling via non-canonical activation of vascular endothelial growth factor receptors (VEGFRs)^48^,^49^. In this regard, we observed an increased number of testicular blood vessels in EAO lesions, which was associated with the severity of the disease and was lower in *Lgals1*^*−/−*^ mice versus WT mice with the same EAO score (Supplementary Fig. S3 online).

Nevertheless, administration of exogenous rGal-1 suppressed the clinical signs of EAO and dampened testicular inflammation. This inhibitory effect followed the same anti-inflammatory pattern shown for other models of autoimmune inflammation, including CIA, EAE, experimental autoimmune uveitis and diabetes^30^,^32^,^34^,^35^, supporting its therapeutic use for the treatment of autoimmune testicular inflammation.

In conclusion, our study unveils the dual roles of endogenous versus exogenous Gal-1 in the control of testicular immunopathology. Moreover, our findings highlight the contribution of Gal-1 to germ cell apoptosis and suggest a note of caution regarding the proposed function of Gal-1 in the regulation of testicular immune privilege. Finally, our studies validate the relevance of Gal-1 as a therapeutic agent for the treatment of testicular immunopathology.

## Additional Information

**How to cite this article**: Pérez, C. V. *et al*. Dual roles of endogenous and exogenous galectin-1 in the control of testicular immunopathology. *Sci. Rep*. **5**, 12259; doi: 10.1038/srep12259 (2015).

## Supplementary Material

Supplementary Information
