# Supplementary material for: Dual roles of endogenous and exogenous galectin-1 in the control of testicular immunopathology
Source: Sci Rep. 2015 Jul 30;5:12259. doi: 10.1038/srep12259 (PMC4519738; doi:10.1038/srep12259)
Supplement: Supplementary Information [file srep12259-s1.pdf]

Dual roles of endogenous and exogenous galectin-1 in the control of testicular immunopathology

Cecilia V. Pérez[1], Leticia G. Gómez[1], Gisela S. Gualdoni[1], Livia Lustig[1], Gabriel A. Rabinovich[2,3], and Vanesa A. Guazzone[1*]

Supplementary Fig. S1 online

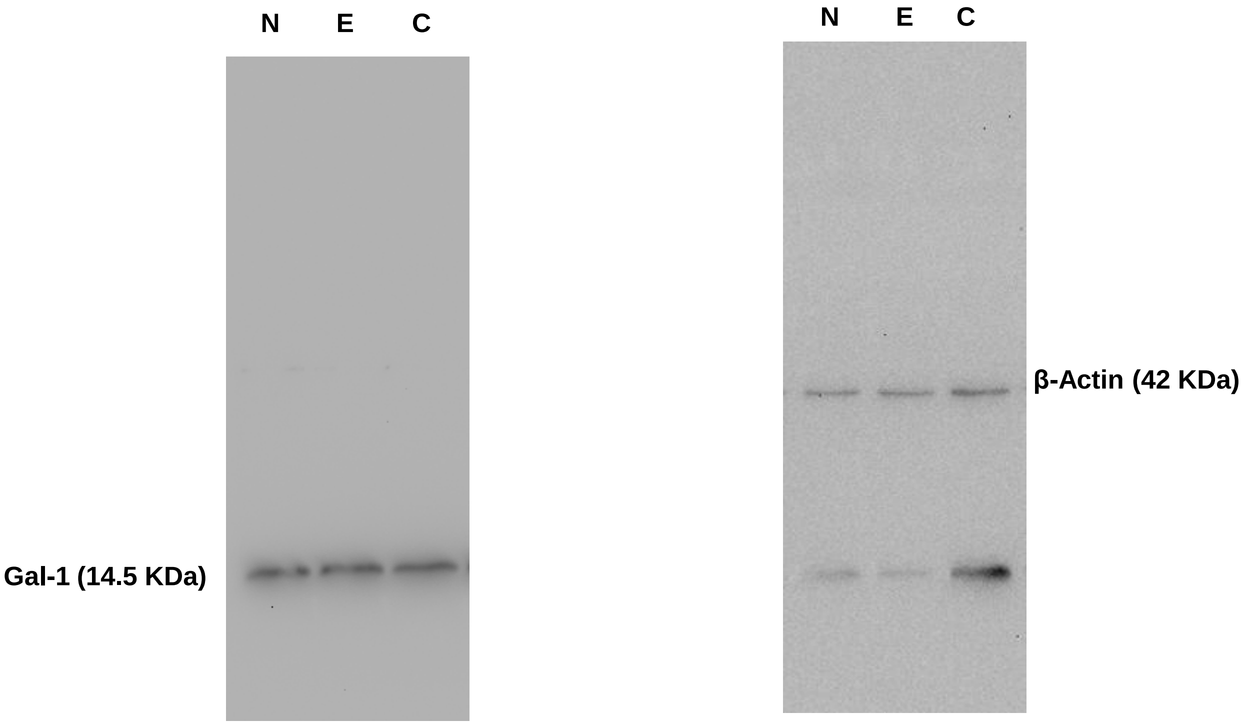

Supplementary Fig. S2 online

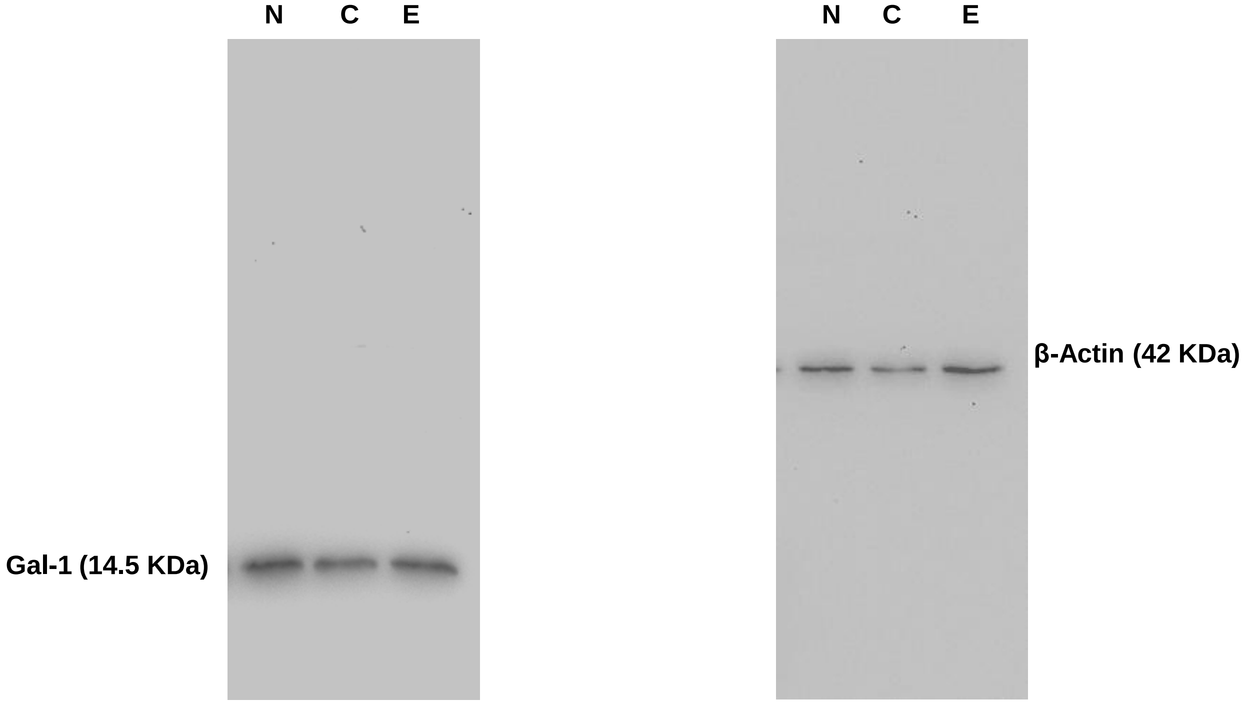

Supplementary Fig. S3 online

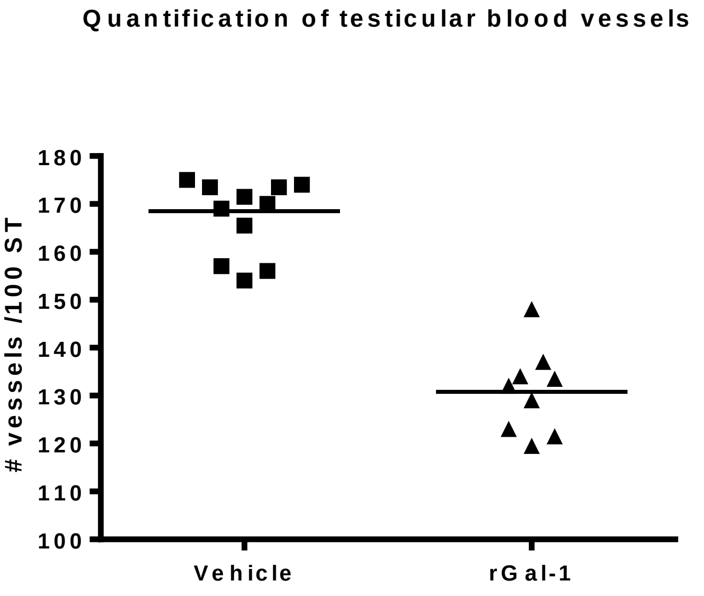

Quantification of blood vessel in testis of mice immunized with testicular homogenate and adjuvants and injected with saline solution (vehicle) or recombinant-Galectin-1 (rGal-1). ST: seminiferous tubules
